# [μ-1,2-Bis(4-pyrid­yl)ethane-κ^2^ *N*:*N*′]bis­[(4′-phenyl-2,2′:6′,2′′-terpyridine-κ^3^ *N*,*N*′,*N*′′)silver(I)] bis­(trifluoro­methane­sulfonate)

**DOI:** 10.1107/S1600536810031776

**Published:** 2010-08-18

**Authors:** Yajun Ma, Buming Liu, Chenghu Xue

**Affiliations:** a School of Chemistry and Chemical Engineering, Yu Lin University, Yulin 719000, People’s Republic of China

## Abstract

In the title compound, [Ag_2_(C_12_H_12_N_2_)(C_21_H_15_N_3_)_2_](CF_3_SO_3_)_2_, the Ag^I^ atom is coordinated by three N atoms of one 4′-phenyl-2,2′:6′,2′′-terpyridine (phtpy) ligand and one pyridyl N atom of the 1,2-bis­(4-pyrid­yl)ethane (bpe) ligand, displaying a distorted square-planar geometry. Two Ag^I^ atoms are bridged by one *trans*-bpe ligand, generating a dinuclear cation. The dinuclear cation is located on a centre of inversion, which is in the middle of the ethyl­ene fragment of the bpe ligand. In the crystal, the pyridyl rings of neighboring dinuclear units are stacked by π–π inter­actions with centroid–centroid distances of 3.667 (2) and 3.835 (2) Å. The F and O atoms of the CF_3_SO_3_^−^ anions are involved in inter­molecular C—H⋯F and C—H⋯O hydrogen-bonding inter­actions, respectively, with –CH groups from the phtpy ligands.

## Related literature

For related complexes with phtpy as a ligand, see: Chen *et al.* (2005 ▶); Constable *et al.* (1990 ▶); Hou & Li (2005 ▶); Rao *et al.* (1997 ▶); Shi *et al.* (2007 ▶); Tu *et al.* (2004 ▶); Xie *et al.* (2008 ▶).
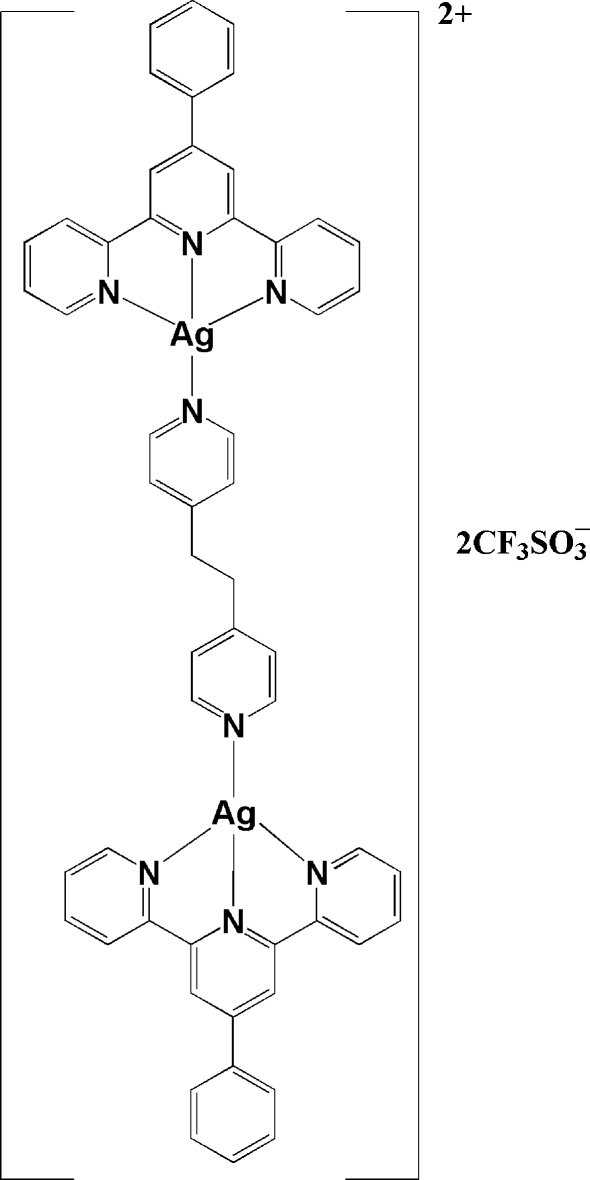


## Experimental

### 

#### Crystal data

[Ag_2_(C_12_H_12_N_2_)(C_21_H_15_N_3_)_2_](CF_3_O_3_S)_2_
*M* *_r_* = 1316.86
Monoclinic, 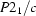

*a* = 7.8345 (8) Å
*b* = 17.4048 (17) Å
*c* = 20.5608 (18) Å
β = 108.795 (3)°
*V* = 2654.1 (4) Å^3^
*Z* = 2
Mo *K*α radiation
μ = 0.90 mm^−1^
*T* = 295 K
0.18 × 0.12 × 0.10 mm

#### Data collection

Bruker SMART APEX area-detector diffractometer
Absorption correction: multi-scan (*SADABS*; Sheldrick, 1996 ▶) *T*_min_ = 0.855, *T*_max_ = 0.916
14927 measured reflections
5194 independent reflections
4170 reflections with *I* > 2σ(*I*)
*R*_int_ = 0.022

#### Refinement

*R*[*F* ^2^ > 2σ(*F* ^2^)] = 0.040
*wR*(*F* ^2^) = 0.119
*S* = 1.08
5194 reflections
361 parameters
H-atom parameters constrained
Δρ_max_ = 0.54 e Å^−3^
Δρ_min_ = −0.50 e Å^−3^

### 

Data collection: *SMART* (Bruker, 2002 ▶); cell refinement: *SAINT* (Bruker, 2002 ▶); data reduction: *SAINT*; program(s) used to solve structure: *SHELXS97* (Sheldrick, 2008 ▶); program(s) used to refine structure: *SHELXL97* (Sheldrick, 2008 ▶); molecular graphics: *SHELXTL* (Sheldrick, 2008 ▶); software used to prepare material for publication: *SHELXTL*.

## Supplementary Material

Crystal structure: contains datablocks I, global. DOI: 10.1107/S1600536810031776/zl2294sup1.cif

Structure factors: contains datablocks I. DOI: 10.1107/S1600536810031776/zl2294Isup2.hkl

Additional supplementary materials:  crystallographic information; 3D view; checkCIF report

## Figures and Tables

**Table 1:** Hydrogen-bond geometry (Å, °)

*D*—H⋯*A*	*D*—H	H⋯*A*	*D*⋯*A*	*D*—H⋯*A*
C12—H12⋯O1^i^	0.93	2.52	3.360 (4)	150
C20—H20⋯F1^i^	0.93	2.56	3.251 (5)	132

Symmetry code: (i) 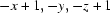.

**Fig. 1.**
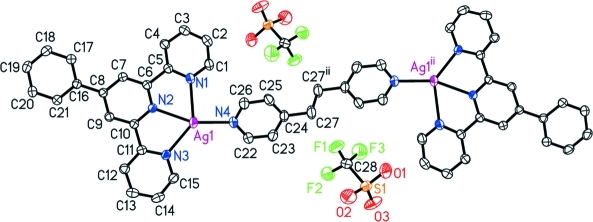
The molecular structure of the title compound, with displacement ellipsoids drawn at the 30% probability level, and H atoms were omitted for clarity; symmetry code, ii: 2-x, 1-y, 1-z.

**Fig. 2.**
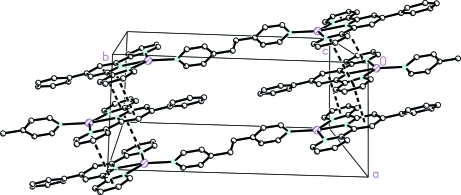
Packing diagram of the title compound showing the intermolecular π–π stacking interactions. The H atoms and CF3SO3- anions have been omitted for clarity.

**Fig. 3.**
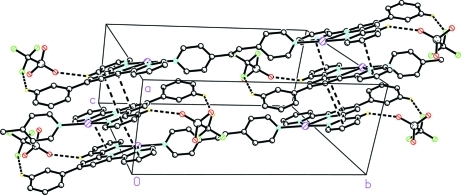
Packing diagram of the title compound showing the intermolecular π–π and C-H···F and C-H···O hydrogen bonding interactions. The H atoms not involved in hydrogen bonding have been omitted for clarity.

## supplementary crystallographic information

### Comment

4'-Phenyl-2,2':6',2"-terpyridine (phtpy) is an excellent chelating ligand and arising from its good coordinating abilities with a broad variety of transition metal ions, such as Cu^I^, Ag^I^, Mn^II^, Ni^II^, Cu^II^, Zn^II^ and Ru^II^ metal ions, it has recently been the focus of several investigations (Chen *et al.*, 2005; Constable *et al.*, 1990; Hou & Li, 2005; Rao *et al.*, 1997; Shi *et al.*, 2007; Tu *et al.*, 2004; Xie *et al.*, 2008). Some of the reported complexes exhibit interesting photoluminescent and magnetic properties. We report here the synthesis and crystal structure of a new Ag^I^ complex incorporating both phtpy and 1,2-bis(4-pyridy)ethane (bpe) as ligands.

In the title compound, [Ag_2_(C_12_H_12_N_2_)(C_21_H_15_N_3_)_2_] (CF_3_SO_3_)_2_, the asymmetric unit is composed of one Ag atom, one phtpy ligand, one half bpe ligand and one CF_3_SO_3_^-^ anion, as shown in Fig. 1. The Ag^I^ centre is four-coordinated by three N atoms of one phtpy ligand and one pyridyl N atom of the bpe ligand, resulting in a distorted square-planar geometry. The sum of the angles about the Ag^I^ centre is 385.02°. The bpe ligand exhibits a *trans*-mode at the ethylene unit (C24/C27/C27^ii^/C24^ii^, symmetry code, ii = 2-x, 1-y, 1-z) and bridges the two Ag^I^ centres to generate a dinuclear structure, which is located on a center of inversion in the middle of the ethylene group of the bpe ligand. In the solid state, the phtpy ligands are π-stacking with phtpy units from neighboring complexes so as to form stacks along the *a*-axis of the cell. Alternating phtpy units in the stacks have opposite orientation with one phtpy unit of each dinuclear complex being part of one stack, while the other phtpy unit is part of the next neighboring stack. The interdigitating π-stacked columns thus form layers of connected stacks that stretch perpendicular to the c-axis of the unit cell. Closest entroid-to-centroid distances within the stacks are of 3.835 (2) Å and 3.667 (2) Å for the distances of the N3 pyridyl and the N1^iii^ and N2^i^ pyridyl rings, respectively (Fig. 2). (Symmetry operators: i = 1-x, -y, 1-z, iii = 2-x, -y, 1-z). In addition, the F1 and O1 atoms of CF_3_SO_3_^-^ anions are involved in intermolecular C-H···F and C-H···O hydrogen bonding interactions, respectively, with -CH groups from the phtpy ligands (Fig. 3).

### Experimental

The ligand 4'-phenyl-2,2':6',2"-terpyridine (phtpy) was synthesized according to the documented method (Constable *et al.*, 1990). A mixture of silver trifluoromethanesulfonate (0.05 mmol, 12.8 mg), phtpy (0.1 mmol, 30.9 mg), 1,2-bis(4-pyridy)ethane (0.05 mmol, 9.2 mg) and 8 ml acetonitrile was stirred in a 25 ml beaker at room temperature for 3 h. After filtration, the filtrate was placed at room temperature for one week to give yellow prismatic crystals of the title compound. Yield: 22.4 mg (34 %). Mp: above 573 K. Anal. C, 51.08; H, 3.21; N, 8.51 %. Found: C, 51.12; H, 3.24; N, 8.46 %. IR (KBr, cm^-1^): 3061, 2925, 1601, 1472, 1408, 1301, 1252, 1015, 875.

### Refinement

The carbon-bound H atoms were placed at calculated positions (C—H = 0.93 Å or 0.97 Å) and refined as riding, with *U*(H) = 1.2*U*_eq_(C), for aryl and ethylene H atoms, respectively.

### Crystal data

**Table:**

[Ag_2_(C_12_H_12_N_2_)(C_21_H_15_N_3_)_2_](CF_3_O_3_S)_2_	*F*(000) = 1324
*M**_r_* = 1316.86	*D*_x_ = 1.648 Mg m^−^^3^
Monoclinic, *P*2_1_/*c*	Mo *K*α radiation, λ = 0.71073 Å
Hall symbol: -P 2ybc	Cell parameters from 4745 reflections
*a* = 7.8345 (8) Å	θ = 2.3–25.0°
*b* = 17.4048 (17) Å	µ = 0.90 mm^−^^1^
*c* = 20.5608 (18) Å	*T* = 295 K
β = 108.795 (3)°	Prism, yellow
*V* = 2654.1 (4) Å^3^	0.18 × 0.12 × 0.10 mm
*Z* = 2	

### Data collection

**Table:**

Bruker SMART APEX area-detector diffractometer	5194 independent reflections
Radiation source: fine-focus sealed tube	4170 reflections with *I* > 2σ(*I*)
graphite	*R*_int_ = 0.022
φ and ω scans	θ_max_ = 26.0°, θ_min_ = 1.6°
Absorption correction: multi-scan (*SADABS*; Sheldrick, 1996)	*h* = −9→9
*T*_min_ = 0.855, *T*_max_ = 0.916	*k* = −21→21
14927 measured reflections	*l* = −25→25

### Refinement

**Table:**

Refinement on *F*^2^	Primary atom site location: structure-invariant direct methods
Least-squares matrix: full	Secondary atom site location: difference Fourier map
*R*[*F*^2^ > 2σ(*F*^2^)] = 0.040	Hydrogen site location: inferred from neighbouring sites
*wR*(*F*^2^) = 0.119	H-atom parameters constrained
*S* = 1.08	*w* = 1/[σ^2^(*F*_o_^2^) + (0.0657*P*)^2^ + 0.7584*P*] where *P* = (*F*_o_^2^ + 2*F*_c_^2^)/3
5194 reflections	(Δ/σ)_max_ = 0.002
361 parameters	Δρ_max_ = 0.54 e Å^−^^3^
0 restraints	Δρ_min_ = −0.50 e Å^−^^3^

### Special details

**Table:**

Geometry. All esds (except the esd in the dihedral angle between two l.s. planes) are estimated using the full covariance matrix. The cell esds are taken into account individually in the estimation of esds in distances, angles and torsion angles; correlations between esds in cell parameters are only used when they are defined by crystal symmetry. An approximate (isotropic) treatment of cell esds is used for estimating esds involving l.s. planes.
Refinement. Refinement of F^2^ against ALL reflections. The weighted R-factor wR and goodness of fit S are based on F^2^, conventional R-factors R are based on F, with F set to zero for negative F^2^. The threshold expression of F^2^ > 2sigma(F^2^) is used only for calculating R-factors(gt) etc. and is not relevant to the choice of reflections for refinement. R-factors based on F^2^ are statistically about twice as large as those based on F, and R- factors based on ALL data will be even larger.

### Fractional atomic coordinates and isotropic or equivalent isotropic displacement parameters (Å^2^)

**Table:**

	*x*	*y*	*z*	*U*_iso_*/*U*_eq_	
Ag1	0.83117 (4)	0.124375 (13)	0.540004 (13)	0.06447 (14)	
N1	0.8764 (4)	0.10707 (15)	0.42457 (14)	0.0530 (6)	
N2	0.7513 (3)	0.00035 (13)	0.49378 (11)	0.0402 (5)	
N3	0.7565 (3)	0.04668 (14)	0.61909 (12)	0.0484 (6)	
N4	0.8557 (4)	0.25065 (15)	0.54219 (14)	0.0568 (7)	
C1	0.9334 (5)	0.1619 (2)	0.39122 (19)	0.0605 (8)	
H1	0.9685	0.2087	0.4133	0.073*	
C2	0.9433 (5)	0.1533 (2)	0.32641 (18)	0.0598 (8)	
H2	0.9841	0.1930	0.3050	0.072*	
C3	0.8911 (4)	0.0840 (2)	0.29400 (17)	0.0559 (8)	
H3	0.8961	0.0759	0.2499	0.067*	
C4	0.8311 (4)	0.02665 (18)	0.32742 (16)	0.0508 (7)	
H4	0.7944	−0.0204	0.3060	0.061*	
C5	0.8262 (4)	0.03988 (16)	0.39343 (14)	0.0420 (6)	
C6	0.7646 (4)	−0.02060 (16)	0.43270 (14)	0.0400 (6)	
C7	0.7215 (4)	−0.09458 (17)	0.40732 (14)	0.0434 (6)	
H7	0.7368	−0.1086	0.3659	0.052*	
C8	0.6551 (4)	−0.14803 (17)	0.44398 (14)	0.0416 (6)	
C9	0.6437 (4)	−0.12466 (15)	0.50720 (15)	0.0428 (6)	
H9	0.6012	−0.1585	0.5335	0.051*	
C10	0.6957 (4)	−0.05081 (15)	0.53104 (13)	0.0392 (6)	
C11	0.6937 (4)	−0.02467 (16)	0.60008 (14)	0.0413 (6)	
C12	0.6375 (4)	−0.07108 (19)	0.64369 (15)	0.0508 (7)	
H12	0.5959	−0.1205	0.6301	0.061*	
C13	0.6430 (5)	−0.0442 (2)	0.70717 (16)	0.0605 (9)	
H13	0.6055	−0.0751	0.7369	0.073*	
C14	0.7046 (5)	0.0293 (2)	0.72631 (17)	0.0638 (9)	
H14	0.7083	0.0492	0.7687	0.077*	
C15	0.7597 (5)	0.0717 (2)	0.68126 (16)	0.0602 (8)	
H15	0.8023	0.1211	0.6943	0.072*	
C16	0.5957 (4)	−0.22547 (16)	0.41550 (14)	0.0445 (6)	
C17	0.5324 (4)	−0.23678 (18)	0.34475 (16)	0.0529 (7)	
H17	0.5290	−0.1957	0.3154	0.063*	
C18	0.4746 (5)	−0.3084 (2)	0.31780 (18)	0.0636 (9)	
H18	0.4324	−0.3151	0.2704	0.076*	
C19	0.4789 (5)	−0.36933 (19)	0.3599 (2)	0.0645 (9)	
H19	0.4401	−0.4174	0.3413	0.077*	
C20	0.5409 (5)	−0.35943 (19)	0.43023 (18)	0.0620 (9)	
H20	0.5436	−0.4009	0.4591	0.074*	
C21	0.5990 (5)	−0.28787 (18)	0.45772 (16)	0.0549 (8)	
H21	0.6408	−0.2815	0.5052	0.066*	
C22	0.9687 (5)	0.2898 (2)	0.59487 (19)	0.0624 (9)	
H22	1.0267	0.2636	0.6354	0.075*	
C23	1.0020 (6)	0.3663 (2)	0.5915 (2)	0.0690 (10)	
H23	1.0811	0.3910	0.6293	0.083*	
C24	0.9185 (5)	0.40753 (19)	0.5317 (2)	0.0635 (9)	
C25	0.8000 (5)	0.36723 (19)	0.4785 (2)	0.0642 (9)	
H25	0.7381	0.3923	0.4378	0.077*	
C26	0.7729 (5)	0.29046 (19)	0.48535 (18)	0.0595 (8)	
H26	0.6926	0.2647	0.4485	0.071*	
C27	0.9537 (7)	0.4920 (2)	0.5257 (2)	0.0888 (14)	
H27A	0.8399	0.5194	0.5127	0.107*	
H27B	1.0274	0.5111	0.5702	0.107*	
S1	0.31230 (14)	0.32504 (6)	0.30585 (5)	0.0664 (3)	
F1	0.3391 (4)	0.41393 (17)	0.41012 (12)	0.1064 (9)	
F2	0.1381 (4)	0.44991 (16)	0.31985 (14)	0.1094 (9)	
F3	0.0908 (5)	0.3532 (2)	0.37338 (19)	0.1285 (12)	
O1	0.3771 (5)	0.26387 (17)	0.35204 (17)	0.1020 (10)	
O2	0.4462 (6)	0.3727 (2)	0.2939 (2)	0.1197 (14)	
O3	0.1655 (5)	0.3085 (2)	0.24617 (16)	0.1157 (13)	
C28	0.2121 (6)	0.3884 (2)	0.35363 (19)	0.0699 (10)	

### Atomic displacement parameters (Å^2^)

**Table:**

	*U*^11^	*U*^22^	*U*^33^	*U*^12^	*U*^13^	*U*^23^
Ag1	0.0906 (2)	0.03503 (16)	0.0624 (2)	−0.00525 (11)	0.01721 (15)	0.00040 (10)
N1	0.0587 (16)	0.0431 (13)	0.0574 (16)	−0.0039 (12)	0.0192 (13)	0.0038 (12)
N2	0.0405 (13)	0.0391 (12)	0.0383 (12)	0.0027 (10)	0.0087 (10)	0.0011 (10)
N3	0.0579 (15)	0.0387 (13)	0.0437 (13)	0.0068 (11)	0.0097 (12)	−0.0030 (10)
N4	0.0702 (18)	0.0383 (14)	0.0629 (18)	−0.0016 (12)	0.0229 (15)	0.0024 (12)
C1	0.069 (2)	0.0446 (18)	0.069 (2)	−0.0084 (16)	0.0233 (18)	0.0044 (16)
C2	0.058 (2)	0.0544 (19)	0.071 (2)	0.0026 (16)	0.0265 (18)	0.0187 (17)
C3	0.0556 (19)	0.063 (2)	0.0527 (18)	0.0014 (15)	0.0224 (15)	0.0094 (15)
C4	0.0536 (18)	0.0489 (17)	0.0503 (17)	0.0017 (14)	0.0175 (15)	0.0034 (13)
C5	0.0362 (14)	0.0428 (15)	0.0446 (15)	0.0033 (12)	0.0096 (12)	0.0051 (12)
C6	0.0350 (14)	0.0418 (15)	0.0405 (14)	0.0034 (11)	0.0082 (12)	0.0043 (11)
C7	0.0459 (16)	0.0438 (15)	0.0402 (14)	−0.0005 (13)	0.0133 (13)	−0.0010 (12)
C8	0.0417 (15)	0.0416 (14)	0.0409 (14)	0.0001 (11)	0.0125 (12)	−0.0007 (12)
C9	0.0418 (15)	0.0432 (16)	0.0415 (15)	−0.0010 (12)	0.0109 (12)	0.0018 (12)
C10	0.0356 (14)	0.0387 (14)	0.0398 (14)	0.0046 (11)	0.0076 (11)	0.0014 (11)
C11	0.0372 (15)	0.0444 (15)	0.0398 (14)	0.0090 (12)	0.0089 (12)	0.0022 (12)
C12	0.0510 (18)	0.0525 (17)	0.0503 (16)	−0.0020 (14)	0.0184 (14)	−0.0024 (14)
C13	0.059 (2)	0.080 (2)	0.0469 (17)	0.0030 (17)	0.0224 (16)	0.0042 (16)
C14	0.068 (2)	0.077 (2)	0.0449 (17)	0.0126 (18)	0.0164 (16)	−0.0142 (17)
C15	0.078 (2)	0.0487 (18)	0.0493 (18)	0.0056 (16)	0.0140 (17)	−0.0080 (14)
C16	0.0462 (16)	0.0434 (15)	0.0445 (15)	−0.0040 (12)	0.0156 (13)	−0.0028 (12)
C17	0.063 (2)	0.0490 (17)	0.0467 (17)	−0.0053 (15)	0.0183 (15)	−0.0011 (13)
C18	0.074 (2)	0.065 (2)	0.0492 (18)	−0.0133 (17)	0.0163 (17)	−0.0135 (16)
C19	0.078 (2)	0.0494 (19)	0.066 (2)	−0.0161 (16)	0.0222 (19)	−0.0162 (16)
C20	0.083 (3)	0.0430 (17)	0.063 (2)	−0.0105 (16)	0.0269 (19)	−0.0012 (15)
C21	0.071 (2)	0.0472 (17)	0.0462 (17)	−0.0063 (15)	0.0181 (15)	−0.0018 (13)
C22	0.076 (2)	0.0504 (19)	0.062 (2)	0.0000 (17)	0.0233 (18)	0.0037 (16)
C23	0.084 (3)	0.054 (2)	0.074 (2)	−0.0155 (18)	0.033 (2)	−0.0126 (18)
C24	0.087 (3)	0.0404 (17)	0.079 (2)	−0.0082 (17)	0.049 (2)	−0.0047 (16)
C25	0.076 (2)	0.0498 (19)	0.070 (2)	0.0067 (17)	0.028 (2)	0.0167 (16)
C26	0.060 (2)	0.0495 (18)	0.066 (2)	−0.0042 (15)	0.0171 (17)	0.0005 (16)
C27	0.140 (4)	0.043 (2)	0.111 (3)	−0.012 (2)	0.079 (3)	−0.002 (2)
S1	0.0776 (6)	0.0679 (6)	0.0576 (5)	−0.0045 (5)	0.0271 (5)	−0.0090 (4)
F1	0.135 (2)	0.109 (2)	0.0634 (14)	0.0154 (18)	0.0157 (15)	−0.0260 (14)
F2	0.151 (3)	0.0865 (18)	0.0858 (17)	0.0446 (17)	0.0304 (17)	0.0173 (14)
F3	0.143 (3)	0.121 (2)	0.168 (3)	−0.009 (2)	0.115 (3)	−0.007 (2)
O1	0.134 (3)	0.0624 (18)	0.104 (2)	0.0260 (18)	0.032 (2)	0.0056 (16)
O2	0.133 (3)	0.129 (3)	0.134 (3)	−0.035 (2)	0.094 (3)	−0.016 (2)
O3	0.108 (2)	0.154 (3)	0.0690 (19)	0.007 (2)	0.0062 (18)	−0.049 (2)
C28	0.097 (3)	0.064 (2)	0.050 (2)	0.003 (2)	0.026 (2)	0.0051 (17)

### Geometric parameters (Å, °)

**Table:**

Ag1—N4	2.205 (3)	C14—C15	1.360 (5)
Ag1—N3	2.330 (3)	C14—H14	0.9300
Ag1—N2	2.360 (2)	C15—H15	0.9300
Ag1—N1	2.528 (3)	C16—C21	1.386 (4)
N1—C5	1.331 (4)	C16—C17	1.391 (4)
N1—C1	1.333 (4)	C17—C18	1.379 (5)
N2—C10	1.336 (4)	C17—H17	0.9300
N2—C6	1.344 (4)	C18—C19	1.363 (5)
N3—C15	1.343 (4)	C18—H18	0.9300
N3—C11	1.346 (4)	C19—C20	1.380 (5)
N4—C26	1.334 (4)	C19—H19	0.9300
N4—C22	1.343 (4)	C20—C21	1.383 (4)
C1—C2	1.368 (5)	C20—H20	0.9300
C1—H1	0.9300	C21—H21	0.9300
C2—C3	1.374 (5)	C22—C23	1.363 (5)
C2—H2	0.9300	C22—H22	0.9300
C3—C4	1.377 (4)	C23—C24	1.392 (5)
C3—H3	0.9300	C23—H23	0.9300
C4—C5	1.389 (4)	C24—C25	1.376 (5)
C4—H4	0.9300	C24—C27	1.507 (5)
C5—C6	1.498 (4)	C25—C26	1.367 (5)
C6—C7	1.390 (4)	C25—H25	0.9300
C7—C8	1.399 (4)	C26—H26	0.9300
C7—H7	0.9300	C27—C27^i^	1.487 (8)
C8—C9	1.392 (4)	C27—H27A	0.9700
C8—C16	1.483 (4)	C27—H27B	0.9700
C9—C10	1.389 (4)	S1—O1	1.408 (3)
C9—H9	0.9300	S1—O3	1.415 (3)
C10—C11	1.496 (4)	S1—O2	1.420 (3)
C11—C12	1.380 (4)	S1—C28	1.815 (4)
C12—C13	1.374 (4)	F1—C28	1.338 (5)
C12—H12	0.9300	F2—C28	1.305 (4)
C13—C14	1.378 (5)	F3—C28	1.301 (5)
C13—H13	0.9300		
			
N4—Ag1—N3	127.16 (9)	C15—C14—C13	117.9 (3)
N4—Ag1—N2	158.38 (9)	C15—C14—H14	121.0
N3—Ag1—N2	69.91 (8)	C13—C14—H14	121.0
N4—Ag1—N1	95.75 (9)	N3—C15—C14	124.0 (3)
N3—Ag1—N1	136.79 (8)	N3—C15—H15	118.0
N2—Ag1—N1	67.19 (8)	C14—C15—H15	118.0
C5—N1—C1	118.5 (3)	C21—C16—C17	118.2 (3)
C5—N1—Ag1	116.58 (19)	C21—C16—C8	121.7 (3)
C1—N1—Ag1	124.7 (2)	C17—C16—C8	120.1 (3)
C10—N2—C6	119.5 (2)	C18—C17—C16	120.5 (3)
C10—N2—Ag1	118.15 (17)	C18—C17—H17	119.7
C6—N2—Ag1	122.28 (18)	C16—C17—H17	119.7
C15—N3—C11	118.0 (3)	C19—C18—C17	120.7 (3)
C15—N3—Ag1	122.9 (2)	C19—C18—H18	119.7
C11—N3—Ag1	118.89 (18)	C17—C18—H18	119.7
C26—N4—C22	116.9 (3)	C18—C19—C20	119.8 (3)
C26—N4—Ag1	119.0 (2)	C18—C19—H19	120.1
C22—N4—Ag1	123.5 (2)	C20—C19—H19	120.1
N1—C1—C2	123.8 (3)	C19—C20—C21	120.0 (3)
N1—C1—H1	118.1	C19—C20—H20	120.0
C2—C1—H1	118.1	C21—C20—H20	120.0
C1—C2—C3	117.8 (3)	C20—C21—C16	120.8 (3)
C1—C2—H2	121.1	C20—C21—H21	119.6
C3—C2—H2	121.1	C16—C21—H21	119.6
C2—C3—C4	119.3 (3)	N4—C22—C23	122.8 (4)
C2—C3—H3	120.3	N4—C22—H22	118.6
C4—C3—H3	120.3	C23—C22—H22	118.6
C3—C4—C5	119.2 (3)	C22—C23—C24	120.4 (4)
C3—C4—H4	120.4	C22—C23—H23	119.8
C5—C4—H4	120.4	C24—C23—H23	119.8
N1—C5—C4	121.3 (3)	C25—C24—C23	116.3 (3)
N1—C5—C6	117.1 (3)	C25—C24—C27	121.8 (4)
C4—C5—C6	121.7 (3)	C23—C24—C27	121.9 (4)
N2—C6—C7	121.3 (3)	C26—C25—C24	120.3 (3)
N2—C6—C5	116.7 (2)	C26—C25—H25	119.9
C7—C6—C5	122.0 (3)	C24—C25—H25	119.9
C6—C7—C8	120.1 (3)	N4—C26—C25	123.4 (3)
C6—C7—H7	119.9	N4—C26—H26	118.3
C8—C7—H7	119.9	C25—C26—H26	118.3
C9—C8—C7	117.1 (3)	C27^i^—C27—C24	112.7 (4)
C9—C8—C16	121.9 (3)	C27^i^—C27—H27A	109.1
C7—C8—C16	121.0 (3)	C24—C27—H27A	109.1
C10—C9—C8	120.0 (3)	C27^i^—C27—H27B	109.1
C10—C9—H9	120.0	C24—C27—H27B	109.1
C8—C9—H9	120.0	H27A—C27—H27B	107.8
N2—C10—C9	121.8 (3)	O1—S1—O3	116.6 (2)
N2—C10—C11	116.5 (2)	O1—S1—O2	115.6 (3)
C9—C10—C11	121.7 (3)	O3—S1—O2	114.1 (3)
N3—C11—C12	121.0 (3)	O1—S1—C28	102.68 (18)
N3—C11—C10	116.2 (2)	O3—S1—C28	102.7 (2)
C12—C11—C10	122.8 (3)	O2—S1—C28	102.2 (2)
C13—C12—C11	119.9 (3)	F3—C28—F2	108.0 (4)
C13—C12—H12	120.0	F3—C28—F1	107.4 (3)
C11—C12—H12	120.0	F2—C28—F1	105.6 (3)
C12—C13—C14	119.2 (3)	F3—C28—S1	111.7 (3)
C12—C13—H13	120.4	F2—C28—S1	113.9 (3)
C14—C13—H13	120.4	F1—C28—S1	109.9 (3)
			
N4—Ag1—N1—C5	−163.1 (2)	Ag1—N2—C10—C11	−1.4 (3)
N3—Ag1—N1—C5	10.5 (3)	C8—C9—C10—N2	2.4 (4)
N2—Ag1—N1—C5	3.2 (2)	C8—C9—C10—C11	−177.1 (3)
N4—Ag1—N1—C1	11.7 (3)	C15—N3—C11—C12	−1.1 (4)
N3—Ag1—N1—C1	−174.7 (2)	Ag1—N3—C11—C12	−176.1 (2)
N2—Ag1—N1—C1	177.9 (3)	C15—N3—C11—C10	−178.9 (3)
N4—Ag1—N2—C10	−142.1 (3)	Ag1—N3—C11—C10	6.2 (3)
N3—Ag1—N2—C10	3.26 (19)	N2—C10—C11—N3	−3.1 (4)
N1—Ag1—N2—C10	177.9 (2)	C9—C10—C11—N3	176.4 (3)
N4—Ag1—N2—C6	39.7 (4)	N2—C10—C11—C12	179.2 (3)
N3—Ag1—N2—C6	−174.9 (2)	C9—C10—C11—C12	−1.2 (4)
N1—Ag1—N2—C6	−0.24 (19)	N3—C11—C12—C13	0.9 (5)
N4—Ag1—N3—C15	−14.9 (3)	C10—C11—C12—C13	178.5 (3)
N2—Ag1—N3—C15	−179.7 (3)	C11—C12—C13—C14	0.1 (5)
N1—Ag1—N3—C15	173.1 (2)	C12—C13—C14—C15	−0.8 (5)
N4—Ag1—N3—C11	159.7 (2)	C11—N3—C15—C14	0.4 (5)
N2—Ag1—N3—C11	−5.1 (2)	Ag1—N3—C15—C14	175.1 (3)
N1—Ag1—N3—C11	−12.3 (3)	C13—C14—C15—N3	0.5 (6)
N3—Ag1—N4—C26	−131.2 (2)	C9—C8—C16—C21	−27.9 (5)
N2—Ag1—N4—C26	6.7 (4)	C7—C8—C16—C21	153.6 (3)
N1—Ag1—N4—C26	43.2 (3)	C9—C8—C16—C17	151.3 (3)
N3—Ag1—N4—C22	57.8 (3)	C7—C8—C16—C17	−27.2 (4)
N2—Ag1—N4—C22	−164.2 (2)	C21—C16—C17—C18	0.0 (5)
N1—Ag1—N4—C22	−127.7 (3)	C8—C16—C17—C18	−179.1 (3)
C5—N1—C1—C2	0.1 (5)	C16—C17—C18—C19	−0.2 (6)
Ag1—N1—C1—C2	−174.6 (3)	C17—C18—C19—C20	0.2 (6)
N1—C1—C2—C3	0.1 (5)	C18—C19—C20—C21	−0.2 (6)
C1—C2—C3—C4	0.1 (5)	C19—C20—C21—C16	0.1 (6)
C2—C3—C4—C5	−0.5 (5)	C17—C16—C21—C20	0.0 (5)
C1—N1—C5—C4	−0.5 (4)	C8—C16—C21—C20	179.2 (3)
Ag1—N1—C5—C4	174.6 (2)	C26—N4—C22—C23	−1.0 (5)
C1—N1—C5—C6	179.4 (3)	Ag1—N4—C22—C23	170.1 (3)
Ag1—N1—C5—C6	−5.5 (3)	N4—C22—C23—C24	−0.3 (6)
C3—C4—C5—N1	0.7 (5)	C22—C23—C24—C25	1.6 (6)
C3—C4—C5—C6	−179.2 (3)	C22—C23—C24—C27	−179.2 (4)
C10—N2—C6—C7	0.1 (4)	C23—C24—C25—C26	−1.7 (6)
Ag1—N2—C6—C7	178.2 (2)	C27—C24—C25—C26	179.2 (4)
C10—N2—C6—C5	179.5 (2)	C22—N4—C26—C25	1.0 (5)
Ag1—N2—C6—C5	−2.4 (3)	Ag1—N4—C26—C25	−170.5 (3)
N1—C5—C6—N2	5.3 (4)	C24—C25—C26—N4	0.4 (6)
C4—C5—C6—N2	−174.8 (3)	C25—C24—C27—C27^i^	−66.6 (7)
N1—C5—C6—C7	−175.3 (3)	C23—C24—C27—C27^i^	114.3 (6)
C4—C5—C6—C7	4.7 (4)	O1—S1—C28—F3	−56.0 (4)
N2—C6—C7—C8	2.9 (4)	O3—S1—C28—F3	65.4 (4)
C5—C6—C7—C8	−176.5 (3)	O2—S1—C28—F3	−176.1 (3)
C6—C7—C8—C9	−3.1 (4)	O1—S1—C28—F2	−178.7 (3)
C6—C7—C8—C16	175.5 (3)	O3—S1—C28—F2	−57.3 (4)
C7—C8—C9—C10	0.5 (4)	O2—S1—C28—F2	61.2 (4)
C16—C8—C9—C10	−178.0 (3)	O1—S1—C28—F1	63.1 (3)
C6—N2—C10—C9	−2.7 (4)	O3—S1—C28—F1	−175.5 (3)
Ag1—N2—C10—C9	179.0 (2)	O2—S1—C28—F1	−57.1 (3)
C6—N2—C10—C11	176.8 (2)		

Symmetry codes: (i) −*x*+2, −*y*+1, −*z*+1.

### Hydrogen-bond geometry (Å, °)

**Table:**

*D*—H···*A*	*D*—H	H···*A*	*D*···*A*	*D*—H···*A*
C12—H12···O1^ii^	0.93	2.52	3.360 (4)	150.
C20—H20···F1^ii^	0.93	2.56	3.251 (5)	132.

Symmetry codes: (ii) −*x*+1, −*y*, −*z*+1.
